# Genome-Wide SNP Detection, Validation, and Development of an 8K SNP Array for Apple

**DOI:** 10.1371/journal.pone.0031745

**Published:** 2012-02-21

**Authors:** David Chagné, Ross N. Crowhurst, Michela Troggio, Mark W. Davey, Barbara Gilmore, Cindy Lawley, Stijn Vanderzande, Roger P. Hellens, Satish Kumar, Alessandro Cestaro, Riccardo Velasco, Dorrie Main, Jasper D. Rees, Amy Iezzoni, Todd Mockler, Larry Wilhelm, Eric Van de Weg, Susan E. Gardiner, Nahla Bassil, Cameron Peace

**Affiliations:** 1 Plant and Food Research, Palmerston North Research Centre, Palmerston North, New Zealand; 2 Plant and Food Research, Mount Albert Research Centre, Auckland, New Zealand; 3 IASMA Research and Innovation Centre, Foundation Edmund Mach, San Michele all'Adige, Trento, Italy; 4 Laboratory for Fruit Breeding and Biotechnology, Department of Biosystems, Katholieke Universiteit Leuven, Heverlee, Leuven, Belgium; 5 USDA-ARS, National Clonal Germplasm Repository, Corvallis, Oregon, United States of America; 6 Illumina Inc., Hayward, California, United States of America; 7 Plant and Food Research, Hawke's Bay Research Centre, Havelock North, New Zealand; 8 Department of Horticulture and Landscape Architecture, Washington State University, Pullman, Washington, United States of America; 9 Agricultural Research Council, Onderstepoort, South Africa; 10 Department of Horticulture, Michigan State University, East Lansing, Michigan, United States of America; 11 The Donald Danforth Plant Science Center, St. Louis, Missouri, United States of America; 12 Oregon Health Sciences University, Portland, Oregon, United States of America; 13 Plant Breeding, Wageningen University and Research Centre, Wageningen, The Netherlands; Ecole Normale Superieure, France

## Abstract

As high-throughput genetic marker screening systems are essential for a range of genetics studies and plant breeding applications, the International RosBREED SNP Consortium (IRSC) has utilized the Illumina Infinium® II system to develop a medium- to high-throughput SNP screening tool for genome-wide evaluation of allelic variation in apple (*Malus*×*domestica*) breeding germplasm. For genome-wide SNP discovery, 27 apple cultivars were chosen to represent worldwide breeding germplasm and re-sequenced at low coverage with the Illumina Genome Analyzer II. Following alignment of these sequences to the whole genome sequence of ‘Golden Delicious’, SNPs were identified using *SoapSNP*. A total of 2,113,120 SNPs were detected, corresponding to one SNP to every 288 bp of the genome. The Illumina GoldenGate® assay was then used to validate a subset of 144 SNPs with a range of characteristics, using a set of 160 apple accessions. This validation assay enabled fine-tuning of the final subset of SNPs for the Illumina Infinium® II system. The set of stringent filtering criteria developed allowed choice of a set of SNPs that not only exhibited an even distribution across the apple genome and a range of minor allele frequencies to ensure utility across germplasm, but also were located in putative exonic regions to maximize genotyping success rate. A total of 7867 apple SNPs was established for the IRSC apple 8K SNP array v1, of which 5554 were polymorphic after evaluation in segregating families and a germplasm collection. This publicly available genomics resource will provide an unprecedented resolution of SNP haplotypes, which will enable marker-locus-trait association discovery, description of the genetic architecture of quantitative traits, investigation of genetic variation (neutral and functional), and genomic selection in apple.

## Introduction

Understanding the links between phenotypic variations and their underlying DNA variation is the major challenge facing plant geneticists today. Recent advances in genomics technologies, including highly parallel sequencing and genetic marker methods for genome-wide assays of allelic variation, have now made high-resolution genetic characterization of crop germplasm feasible. The use of genomics tools has tremendous potential to assist Rosaceae crop breeders to produce significant genetic gains for consumer and grower traits more precisely and efficiently, as well as to improve understanding of the genetic architecture of agronomic characters [1]. Genetic marker-based strategies such as QTL interval mapping, pedigree-based analysis, association mapping, and genomic selection are now powerfully enabled by genomics technologies. Although genetic markers such as Simple Sequence Repeats (SSRs) [2], [3], [4] and Single Nucleotide Polymorphisms (SNPs) [5], [6], [7] have been developed for apple, their density across the genome is not sufficient for fine dissection of functional genetic variation. We focus here on the development of a medium to high-throughput multiplexed SNP assay tool based on the Illumina Infinium® assay for genome-wide evaluation of allelic variation in apple breeding germplasm. This product of international research community collaboration is intended to benefit apple breeders directly, and consequently growers and consumers who demand better quality pome fruit that is produced more sustainably.

SNP marker development for a genome screening tool is based on three steps: detection, validation, and final selection. The objectives and throughput required are different for each step. The detection step requires identification of a large pool of SNPs in the crop and typically involves non-targeted techniques such as re-sequencing, or High Resolution Melting (HRM) [8], of a few representative yet diverse individuals. This maximizes opportunities for detecting SNPs, while ensuring that the SNPs will be suitable for their final application for screening breeding germplasm. While sequencing has historically been carried out using the Sanger method, high-throughput sequencing-by-synthesis techniques have now made it possible to re-sequence entire genomes affordably [9]. Although not strictly necessary, validation is desirable to inform SNP assay development, in order to maximize the number of functional polymorphic markers in the final genetic analysis. Because of the current prohibitive cost of validating every detected SNP, validation is often based on a subset of the detected SNPs that are screened over an informative set of individuals. If a range of SNP filtering parameter values are included in the SNPs used for the validation step, suitable parameter thresholds can then be established for those SNPs to be selected for the final assay. For development of genome-wide SNP assays, adequate genome coverage by the final set chosen is critical [10]. In crops with whole genome sequences and saturated genetic maps, such as apple [11], genome coverage can be on a physical and/or a genetic basis, as desired. When such genomics resources are lacking, the assumption may be made that random distribution of a large number of SNPs will achieve adequate coverage, with the caveat that certain genomic regions will be over- or under-represented depending on the SNP detection approach adopted.

Once SNP sets have been developed, screening can be carried out with a range of highly parallel techniques on segregating germplasm sets according to the research need or breeding application. Multiplexing and automation have enhanced the efficiency of SNP genotyping enormously and several high-throughput platforms are now available for the genotyping of a variable number of samples for one to up to one million SNPs in parallel. They include, but are not limited to, TaqMan® from Applied Biosystems and array-based technologies from Illumina (GoldenGate® and Infinium®) or Affymetrix [10], [12], [13].

We used next-generation sequencing (NGS) to detect SNPs covering the apple genome. This effort involved re-sequencing a small set of cultivars, ancestors, and founders, chosen to represent the pedigrees of worldwide apple breeding programs by RosBREED, a consortium established to enable marker-assisted breeding for Rosaceae crops (www.rosbreed.org; [14]). We validated the SNPs detected and determined adequate filtering parameter values, using the Illumina GoldenGate® assay to screen a larger set of accessions from the international apple breeding germplasm. Based on results of the GoldenGate® assay, we further refined SNP-filtering criteria to enable the development of an 8K Infinium® II array and evaluated it, using a segregating population of apple seedlings.

## Materials and Methods

**Figure 1:**
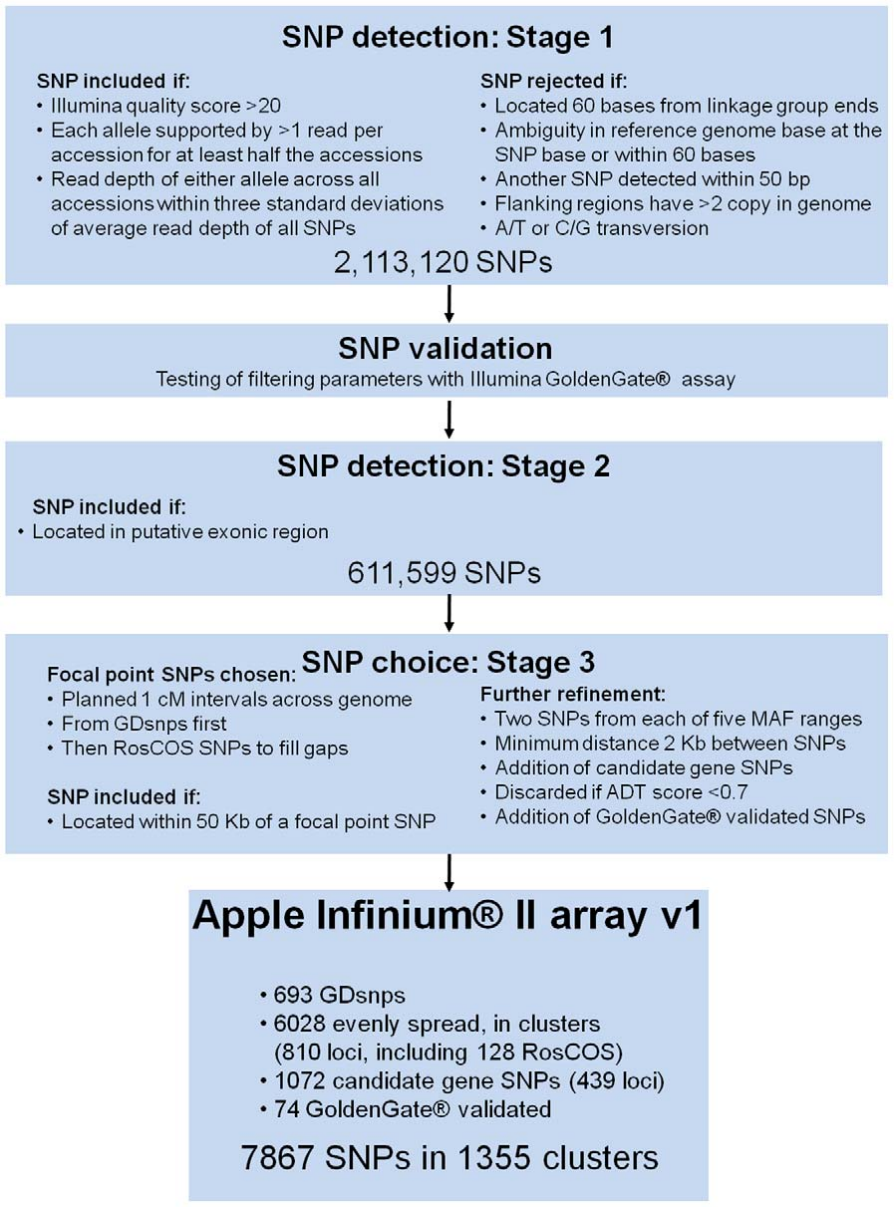
Workflow for single nucleotide polymorphism (SNP) detection, validation, and final choice employed for development of the IRSC apple 8K SNP array v1. GDsnp: ‘Golden Delicious’-validated SNP; RosCOS: Rosaceae Conserved Orthologous Set; MAF: Minor Allele Frequency; ADT: Assay Design Tool.

The workflow and design parameters described below are summarized in Figure 1.

### Whole genome re-sequencing of apple breeding accessions

A SNP detection panel of 27 apple germplasm accessions was chosen for whole-genome, low coverage re-sequencing. The accessions were founders, intermediate ancestors, or important breeding parents used extensively in apple breeding programs worldwide. The complete set maximized coverage of the genetic background of the world's cultivated apple. Individuals in the set were *Malus×domestica* ‘Braeburn’, Co-op 15, ‘Cox's Orange Pippin’, ‘CrimsonCrisp’, ‘Cripps Pink’, ‘Delicious’ (both the original form and the derived mutant ‘Red Delicious’), ‘Dolgo’, ‘Duchess of Oldenburg’, F_2_26829-2-2, ‘Frostbite’, ‘Fuji’, ‘Geneva’, ‘Golden Delicious’, ‘Granny Smith’, ‘Haralson’, ‘Honeycrisp’, ‘Idared’, ‘James Grieve’, ‘Jonathan’, ‘McIntosh’, ‘Ralls Janet’, ‘Red Dougherty’, ‘Rome Beauty’, ‘Splendour’, ‘Zestar’, and *Malus sieversii* PI613981. Each accession was sequenced using one lane of Illumina GA II (except for ‘Cox's Orange Pippin’ that was sequenced twice) with 75 to 80 cycles per read and small insert paired-end or single-end sequencing as specified in Table 1. The raw sequenced data were retrieved each apple accession and then aligned separately to the reference genome of ‘Golden Delicious’ [11] using *Soap2* [15].

**Table 1:** Apple cultivars used for low coverage re-sequencing and subsequent single nucleotide polymorphism detection.

Accession	Number of reads	Type of reads	Estimated genome coverage (X)	Location of sequencing
‘Braeburn’	34,699,040	Paired-end	6.9	PFR
‘Co-op 15’	51,864,610	Single-end	5.2	ARC
‘Cox's Orange Pippin’	35,120,961	Paired-end	7.0	PFR & ARC
‘Crimson Crisp’	6,128,575	Paired-end	1.3	RosBREED
‘Cripps Pink’	56,738,286	Single-end	6.1	ARC
‘Delicious’	17,995,052	Paired-end	1.9	RosBREED
‘Dolgo’	59,863,282	Single-end	6.4	ARC
‘Duchess of Oldenburg’	17,762,643	Paired-end	3.8	RosBREED
F_2_26829-2-2	31,580,732	Paired-end	6.7	RosBREED
‘Frostbite’	44,740,552	Single-end	4.5	ARC
‘Fuji’	44,545,764	Single-end	4.8	ARC
‘Geneva’	31,977,525	Paired-end	6.4	PFR
‘Golden Delicious’	22,101,159	Paired-end	4.7	RosBREED
‘Granny Smith’	36,112,204	Paired-end	7.2	PFR
‘Haralson’	15,499,148	Paired-end	3.3	RosBREED
‘Honeycrisp’	20,887,451	Paired-end	4.5	RosBREED
‘Idared’	33,479,444	Paired-end	6.7	PFR
‘James Grieve’	26,005,304	Paired-end	5.2	PFR
‘Jonathan’	18,812,375	Paired-end	4.0	RosBREED
‘McIntosh’	59,934,507	Paired-end	12.8	RosBREED
*Malus sieversii* PI613981	23,500,375	Paired-end	4.7	USDA-ARS
‘Ralls Janet’	35,485,026	Paired-end	7.1	PFR
‘Red Delicious’	25,993,982	Paired-end	5.2	PFR
‘Red Dougherty’	32,718,666	Paired-end	6.5	PFR
‘Rome Beauty’	23,220,046	Paired-end	5.0	RosBREED
‘Splendour’	36,353,918	Paired-end	7.3	PFR
‘Zestar’	54,404,792	Single-end	5.4	ARC

An approximate genome coverage was estimated for each accession using a genome size of 750 Mb. The source of cultivars and numbers of Illumina GA II reads obtained are indicated. ARC: Agricultural Research Council, South Africa; PFR: The New Zealand Institute for Plant & Food Research Ltd; RosBREED: U.S.-based international project.

### Detection and filtering of SNPs

SNPs were detected using *SoapSNP* (http://soap.genomics.org.cn/soapsnp.html) essentially as described by [16], but with the following modifications (Stage 1 filtering): *SoapSNP* calls were discarded if (1) the SNP call was within the first or last 60 bases of a linkage group (LG) sequence, (2) the reference genome base was ambiguous at the SNP position or within 60 bases of it, (3) another SNP was detected within 50 bases, (4) the average copy number of the SNP flanking region was more than two, (5) the Illumina quality score of either allele was less than 20, (6) the number of reads supporting either allele was less than two reads per accession for at least half the accessions, (7) the number of reads for either allele across all accessions was greater than the average read depth of all SNPs plus three standard deviations, and (8) if the call was an A/T or C/G transversion. This filtration yielded “Stage 1 SNPs”.

Stage 1 SNPs were then subjected to a Stage 2 filter, whereby SNPs not located in a predicted exonic region were discarded. For each LG, the exon space was defined by mapping all gene models [11] and 396,643 cDNA sequences from the Plant & Food Research *Malus* Sequence Database libraries [17] (‘AAAA’, ‘AABA’, ‘AACA’, ‘AADA’, ‘AAEA’, ‘AAMA’, ‘AAFA’, ‘AAFB’, ‘AAGA’, ‘AAHA’, ‘AAIA’, ‘AAJA’, ‘AAKA’, ‘AALA’, ‘AALB’, ‘AANA’, ‘AAOA’, ‘AAPA’, ‘AAQA’, ‘AARA’, ‘AASA’, ‘AAUA’, ‘AAVB’, ‘AVBC’, ‘AAWA’, ‘AAXA’, ‘AAYA’, ‘AAZA’, ‘AATA’, ‘ABAA’, ‘ABBA’, ‘AASB’, ‘AASC’, ‘ABBB’, ‘ABAB’, ‘ABDA’, ‘ABCA’, ‘ABEA’, ‘ABHA’, ‘ABKA’, ‘AGDQ’, ‘ABPB’, ‘ABLB’, ‘ABMA’, ‘ABPA’, ‘ABNA’, ‘ABNB’, ‘ABLC’, ‘ABLA’, ‘ABQA’, ‘ABRA’, ‘ABSA’, ‘ABTA’, ‘ABUA’, ‘ABVA’, ‘ABWA’, ‘ABXA’, ‘ABYA’, ‘ABZA’, ‘ACAA’, ‘ACBA’, ‘AELA’, ‘AEPA’, ‘AEAA’, ‘AENA’, ‘AFBB’, ‘AFBA’, ‘AFBC’, ‘AGBM’, ‘AGBN’, ‘AGBO’, ‘AGBP’, ‘AGBQ’, ‘AGBR’, ‘AGBS’, ‘AGBT’, ‘AGBU’, ‘AGBV’, ‘AGBW’, ‘AGCD’, ‘AGCE’, ‘AGCI’, ‘AGCJ’, ‘AGCK’, ‘AGCL’, ‘AGCM’, ‘AGCN’, ‘AGCP’, ‘AGCQ’, ‘AGCR’, ‘AGCS’, ‘AGCT’, ‘AGCU’, ‘AGCV’, ‘AGCW’, ‘AGCX’, ‘AGCY’, ‘AGCZ’, ‘AGDA’, ‘AGDB’, ‘AGDC’, ‘AGDD’, ‘AGDE’, ‘AGDF’, ‘AGDG’, ‘AGDH’, ‘AGDK’, ‘AGDM’, ‘AGDN’, ‘AGDO’, ‘AGDP’, ‘AGDL’, ‘AGDR’, ‘AGDS’, ‘AGDW’, ‘AHAA’, ‘AOFA’, ‘AVBB’, ‘ASYA’, ‘AYFB’, AYFA’) to the reference genome using *gmap* [18] with command line options -B 2 -K 1000 -L 50000 -f 2 -O.

### SNP validation with the GoldenGate® assay

**Figure 2:**
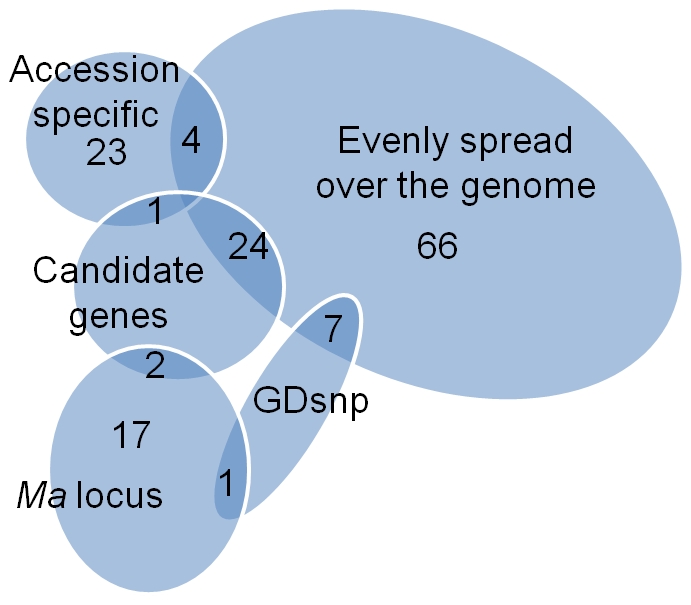
Classification of the 144 apple single nucleotide polymorphisms (SNPs) used for validation using the Illumina GoldenGate® assay.

A subset of 144 SNPs was chosen to validate the efficiency of SNP detection and fine-tune the filtering parameters (Figure 2). The initial selection was of 100 Stage 1 SNPs evenly spread across the apple genome, i.e., the 17 LGs representing the 17 haploid chromosomes of apple, according to the primary assembly of ‘Golden Delicious’ [11]. These included a SNP located in the first 200 kb from each end of each LG. SNPs between these were evenly spaced along the LG every 6-8 Mb, depending on LG length. Spacing across all LGs averaged 7.26 Mb (standard deviation of 0.66 Mb), with a maximum average of 8.01 Mb (±0.07 Mb) for LG2 and a minimum average of 5.84 Mb (±0.36 Mb) for LG16. Twenty more SNPs were targeted to a 1.4 Mb region near the top of LG16, spanning a major trait locus associated with malic acid content in fruit (*Ma* locus; [19]). These *Ma* locus SNPs were spaced an average of 75 Kb apart (ranging from 5 Kb to 186 Kb). It was planned that approximately 20% of the 144 SNPs would be accession-specific, i.e., their minor allele would be detected in only one re-sequenced accession of the detection panel. As only four of the evenly spread SNPs met this criterion, 24 additional SNPs were chosen as accession-specific. These latter SNPs were spread over the genome at 1–2 per LG but otherwise randomly distributed on each LG. Approximately 20% of the 144 SNPs were allocated to meet the criterion of being within exons of candidate genes for fruit quality and plant architectural traits. These included 24 of the evenly spread SNPs, four of the *Ma* locus SNPs, and one of the accession-specific SNPs, totaling 27 exonic candidate gene SNPs. The 144 SNPs also deliberately included a wide range of minor allele frequencies (MAFs) and a wide range of Illumina Assay Design Tool (ADT) scores. Approximately 5% of SNPs were chosen from those previously validated in ‘Golden Delicious’ (GDsnp) using the SNPlex technique [11] and these included seven evenly spread SNPs and one *Ma* locus SNP, to serve as positive controls.

To test the above SNP parameters for their effects on genotyping efficiency, a validation panel of 160 apple accessions (Table S1) was screened with the 144 SNP subset, using the GoldenGate® assay. Individuals in the validation panel are founders, intermediate ancestors, and important breeding parents of modern apple cultivars, forming a complex pedigree structure linking much of the world's cultivated apple crop and incorporating the 27 accessions of the SNP detection panel. The validation panel also included bin-mapping sets of approximately eight seedlings each for: ‘Golden Delicious’×‘Anna’ [20], ‘Malling 9’×‘Robusta 5’ [21], ‘Prima’×‘Fiesta’ [19], ‘Royal Gala’×‘Braeburn’ [11], and ‘Telamon’×‘Braeburn’ [22]. Genomic DNA was purified from each accession using the E-Z 96 Tissue DNA Kit (Omega Bio-Tek, Inc., Norcross, USA). DNA was quantitated with the Quant-iT™ PicoGreen® Assay (Invitrogen, Carlsbad, USA), using the Victor multiplate reader (Perkin Elmer Inc., San Jose, USA). Concentrations were adjusted to a minimum of 50 ng/µl in 5 µl aliquots and were submitted to the Research Technology Support Facility at Michigan State University (http://rtsf.msu.edu/illumina-beadxpress-reader-system), where the GoldenGate® assay was performed following the manufacturer's protocol (Illumina Inc., San Diego, USA). Following amplification, the PCR products were hybridized to VeraCode microbeads via the address sequence for detection on a VeraCode BeadXpress Reader. SNP genotypes were scored with the Genotyping Module of the GenomeStudio Data Analysis software (Illumina Inc.).

### SNP final choice for 8K Infinium® II array

A clustering strategy was devised that would evenly span the genetic map of apple with clusters of exonic SNPs, in order to provide a final SNP genome scan with the capability of determining SNP haplotypes at distinct loci. The design featured focal points at approximately 1 cM intervals, with 4–10 SNPs clustered at each focal point. Choice of focal points began with GDsnps [11] according to their location in the IASMA-FEM ‘Golden Delicious’×‘Scarlet’ reference genetic map (http://genomics.research.iasma.it/cgi-bin/cmap/viewer), followed by Rosaceae Conserved Orthologous Set (RosCOS; [23]) loci to fill genetic gaps between GDsnps, according to physical map location [24]. Chromosome ends, the first 200 Kb of each LG according to the apple draft genome pseudo-chromosomes [11], were also targeted. SNPs not located within ±50 Kb of a focal point SNP were then discarded. Within this pool of candidates, SNPs were binned according to MAF (bins of 0.1, 0.2, 0.3, 0.4, and 0.5 corresponding to MAF ranges of 0.01–0.1, 0.101–0.2, and so on). Two SNPs were chosen from each bin for each cluster, to give a total of up to ten SNPs per cluster (including the focal point SNP). To minimize detection of redundant haplotypes within clusters in subsequent genotyping, the minimum distance between SNPs was set at 2 Kb. Additional SNP clusters were developed from candidate genes for fruit quality, tree architecture, and flowering that were chosen using the available literature [25], [26], [27]. The best hits from a BLAST search of these candidate genes against the apple “Consensus CDS Peptides”, available from the GDR website (http://www.rosaceae.org), created a list of homologous apple sequences that were then located in the apple genome and became new focal points. Stage 2 SNPs identified in these genes were subjected to Stage 3 filtering criteria, except that the “2 Kb minimum distance” rule was relaxed so that multiple SNPs could be chosen within a gene to form a cluster. Instead, up to four SNPs within each candidate gene were manually chosen to lie as far from each other as possible. Finally, SNPs with an ADT score below 0.7 were discarded.

### SNP array evaluation and cluster file development

A set of populations from various crosses, as well as accessions from the apple germplasm, were used to evaluate the Apple 8K Infinium® II array. This set included a ‘Royal Gala’×‘Granny Smith’ F_1_ population of 186 seedlings [28], seven controlled F_1_ crosses that are used as a training population for genomic selection at Plant & Food Research and comprise 1313 individuals [29], and a set of 117 accessions from the Plant & Food Research germplasm collection (S. Kumar, unpublished). Genomic DNA (gDNA) was extracted using the NucleoSpin® Plant II kit (Macherey-Nagel GmbH & Co KG, Düren, Germany), and quantitated using the Quant-iT™ PicoGreen® Assay (Invitrogen). ‘Royal Gala’, ‘Granny Smith’, and ‘Golden Delicious’ were used as controls. Two hundred nanograms of gDNA were used as template for the reaction, following the manufacturer's instructions. SNP genotypes were scored with the Genotyping Module of the GenomeStudio Data Analysis software (Illumina Inc., San Diego, CA). Individuals with low SNP call quality (*p50GC*<0.54), as well as seedlings putatively resulting from an unintended pollination, were removed from the analysis. SNPs with a *GenTrain* score >0.6 were retained and those with scores ranging between 0.3 and 0.6 were visually checked for accuracy of the SNP calling. Clusters were manually edited when the parent-offspring segregation was not correct, or when the number of missing genotypes was greater than 20.

Two trios with both parents and one seedling were used to test the usefulness of SNP clusters for identifying haplotypes: ‘Royal Gala’×‘Braeburn’−>‘Scifresh’ and ‘(Royal) Gala’×‘Splendour’−>‘Sciros’. SNPs were coded using A and B alleles and haplotypes were inferred using FlexQTL™ (www.flexqtl.nl) for each cluster of SNPs.

## Results

### NGS re-sequencing of apple breeding accessions and SNP detection

A total of 67 Gb of DNA sequence from 898 million 75-base reads were generated for the 27 *Malus* accessions (Table 1). The total number of reads per accession ranged from six to 60 million, for ‘Crimson Crisp’ and ‘McIntosh’, respectively. The difference was due to an increase in the cluster density and resulting sequencing yield of the Illumina GA instrument over the period that sequencing was performed. A total of 10,915,756 SNPs was identified using *SoapSNP*, of which 2,113,120 (19.5%) passed the filtering criteria for Stage 1 detection (Table 2, Figure 1). The average SNP frequency was one per 288 bp. Of these SNPs, 611,599 (28.9%) were predicted to be located in exonic regions and passed the Stage 2 filter (Table 2, Figure 1). When the SNP calls based on re-sequencing data for the two independently sequenced ‘Cox's Orange Pippin’ samples were compared, an average of 21.3% of detected SNPs gave different genotypes between the two samples.

**Table 2:** Single nucleotide polymorphisms (SNPs) detected across the apple genome by re-sequencing 27 apple accessions.

Chromosome	Sequence used for SNP detection (bp)	SNPs examined from *SoapSNP*	SNPs passing “Stage 1” filtering	Average distance between SNPs (bp)	Exonic SNPs
1	36,084,648	556,642	102,825	350	31,678
2	40,172,783	793,583	144,715	277	42,496
3	39,907,579	700,701	134,595	296	36,005
4	25,411,901	474,397	91,212	278	27,540
5	37,603,833	661,644	134,672	279	38,400
6	30,670,413	514,972	97,014	316	29,450
7	31,181,013	546,126	117,652	265	33,155
8	35,800,717	625,020	114,173	313	31,946
9	37,514,065	737,124	146,472	256	41,935
10	38,388,612	687,313	146,102	262	43,522
11	40,097,013	751,789	146,718	273	42,681
12	36,276,268	664,326	121,739	297	35,456
13	39,686,055	720,281	158,912	249	43,699
14	34,156,235	607,215	114,259	298	32,712
15	55,775,419	919,506	159,368	349	46,218
16	23,462,870	421,197	85,530	274	24,357
17	27,122,502	533,920	97,162	279	30,349
Total	609,311,926	10,915,756	2,113,120	288	611,599

### SNP validation

In the GoldenGate® validation assay, 148 apple accessions in the test panel gave good quality scores (call rate >0.8 and 10%GC Score >0.5) and 12 accessions failed because of poor DNA quality. Seventy-three (50.7%) of SNPs were polymorphic, 46 (31.9%) had failed reactions, and 25 (17.3%) were monomorphic (MAF <0.05 or A/B frequency <0.1) (Table 3). All eight GDsnps were polymorphic. Evenly spread SNPs and SNPs at the *Ma* locus had similar success rates to the overall set (54% and 55% polymorphic SNPs, respectively). SNPs located within candidate genes were generally more successful than the overall set (63% polymorphic). Of the 28 SNPs chosen on the basis of their accession specificity, 14 (50%) had a MAF <0.05, of which 11 were not monomorphic. The Illumina ADT score calculated for each SNP did not significantly influence the success of the SNPs (Table 3).

**Table 3:** Results from a GoldenGate® assay of 144 single nucleotide polymorphisms (SNPs) screened over 160 apple accessions (Table S1).

		Proportion of SNPs			
SNP type	Total	Failed	Mono-morphic	Polymorphic (MAF<0.05)	Polymorphic (MAF>0.05)
Evenly spread	100	0.32	0.08	0.04	0.56
Accession-specific	28	0.25	0.11	0.39	0.25
Candidate genes	27	0.22	0.11	0.04	0.63
*Ma* locus	20	0.40	0.00	0.05	0.55
GDsnp	8	0.00	0.00	0.00	1.00
ADT score <0.8	40	0.35	0.00	0.05	0.60
ADT score 0.8–0.9	35	0.40	0.06	0.03	0.51
ADT score <0.9	69	0.26	0.12	0.17	0.45
Mean ADT score (s.d.)	0.85 (0.13)	0.83 (0.12)	0.94 (0.04)	0.92 (0.10)	0.83 (0.14)
Total	144	0.32	0.07	0.10	0.51

### SNP final choice

**Figure 3:**
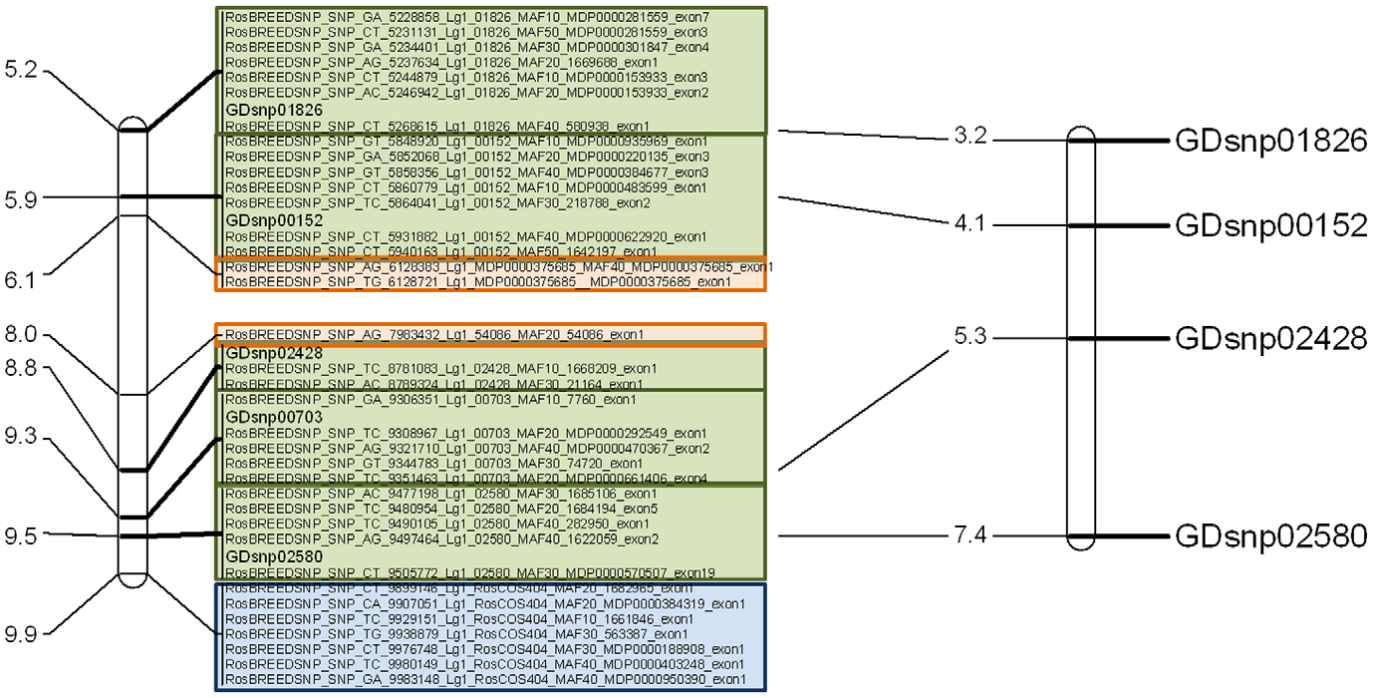
Detailed view of a genomic region at the top of Linkage Group 1 showing SNPs chosen for the International RosBREED SNP Consortium (IRSC) apple 8K SNP array v1. Physical map locations of SNPs (left; in megabases) are compared with known genetic map locations of SNP markers developed from ‘Golden Delicious’ (GDsnp in centiMorgans). SNP clusters around focal points are boxed, with green boxes denoting GDsnps, blue boxes denoting Rosaceae Conserved Orthologous Set markers (RosCOS), and orange boxes denoting candidate genes.

The third stage of filtering, which involved designating focal points at approximate 1 cM intervals over the apple genome and choosing SNPs in 100 Kb windows around them (Figure 3), was initially based on 712 suitable GDsnp markers. These markers left 107 gaps in the genome greater than 3 cM and these gaps were filled using two SSRs and 128 RosCOS as additional focal points, giving a total of 842 focal points. By this stage, 6074 SNPs had been chosen in addition to the initial 712 GDsnps. Next, a further 5528 SNPs were identified in candidate genes and narrowed down to 1652 SNPs after removing putative redundancies. Filtering of the 8438 SNPs chosen by this stage, using the ADT score, reduced the pool to 7793 SNPs: 693 GDsnps, 6028 SNPs around focal points, and 1072 SNPs within candidate genes (Figure 1). Finally, the 74 validated SNPs from the GoldenGate® assay were included, to provide a grand total of 7867 SNPs (Table S2) in 1355 clusters for construction of the final apple Infinium® II SNP array, officially named the International RosBREED SNP Consortium (IRSC) apple 8K SNP array v1.

### The IRSC apple 8K SNP array v1

The 7867 SNPs were uniformly represented on the 17 apple LGs (Table 4). The number of SNP clusters ranged from 64 to 113 on LGs 6 and 2, respectively, with an average of 5.8 SNPs per cluster. The average physical distance between SNP clusters ranged from one cluster every 316.2 kb to 538.3 kb on LG 16 and 3, respectively. The overall cluster density on the ‘Golden Delicious’×‘Scarlet’ reference genetic map was one cluster every centiMorgan, with only five gaps between clusters larger than 10 cM.

**Table 4:** Apple Infinium® II array v1 content and evaluation in the Plant & Food Research dataset. The number of attempted SNPs and clusters is indicated per linkage group (LG), as well as their density based on the apple genome assembly in kilobases (Kb) and the ‘Golden Delicious’ genetic map (cM).

	Attempted SNPs on IRSC Apple Infinium II array v1				Array evaluation in the Plant & Food Research dataset						
LG	No. of SNPs	No. of clusters	Average physical distance between focal points (kb)	Average genetic distance between focal points (cM)	No. of successful beadtypes	No. of poly-morphic SNPs	% poly-morphic SNPs	No. of poly-morphic SNPs with MAF>0.05	MAF>0.05, 50 GC>0.4, call rate >0.95	No. of poly-morphic clusters	% poly-morphic clusters
1	443	81	443.7	1.10	434	299	68.9	250	180	75	92.6
2	695	113	355.1	0.75	684	519	75.9	436	313	108	95.6
3	499	74	538.3	1.19	487	350	71.9	288	212	72	97.3
4	392	65	388.7	1.00	386	292	75.6	235	201	63	96.9
5	492	82	456.9	1.10	486	360	74.1	308	239	76	92.7
6	351	64	473.2	1.17	340	233	68.5	194	117	63	98.4
7	345	71	438.5	0.83	340	215	63.2	181	129	62	87.3
8	405	68	519.9	1.07	399	276	69.2	229	182	62	91.2
9	490	80	449.2	0.91	477	364	76.3	311	232	76	95.0
10	554	83	446.6	1.12	531	377	71.0	271	224	79	95.2
11	466	80	500.7	0.97	456	322	70.6	277	177	73	91.3
12	469	85	426.5	0.87	459	338	73.6	258	210	81	95.3
13	433	81	489.6	0.94	423	299	70.7	229	178	76	93.8
14	388	72	472.2	1.02	374	266	71.1	215	151	64	88.9
15	629	106	525.6	1.09	621	440	70.9	365	253	101	95.3
16	355	74	316.2	0.76	347	271	78.1	215	158	69	93.2
17	461	76	356.7	0.99	448	333	74.3	231	215	70	92.1
Total	7867	1355	446.2	1.00	7692	5554	72.2	4493	3371	1270	93.7

The Apple Infinium® array was evaluated by screening 1398 progeny in 8 segregating populations and 117 individuals from the Plant & Food Research apple germplasm collection.

### IRSC apple 8K SNP array v1 evaluation

**Figure 4:**
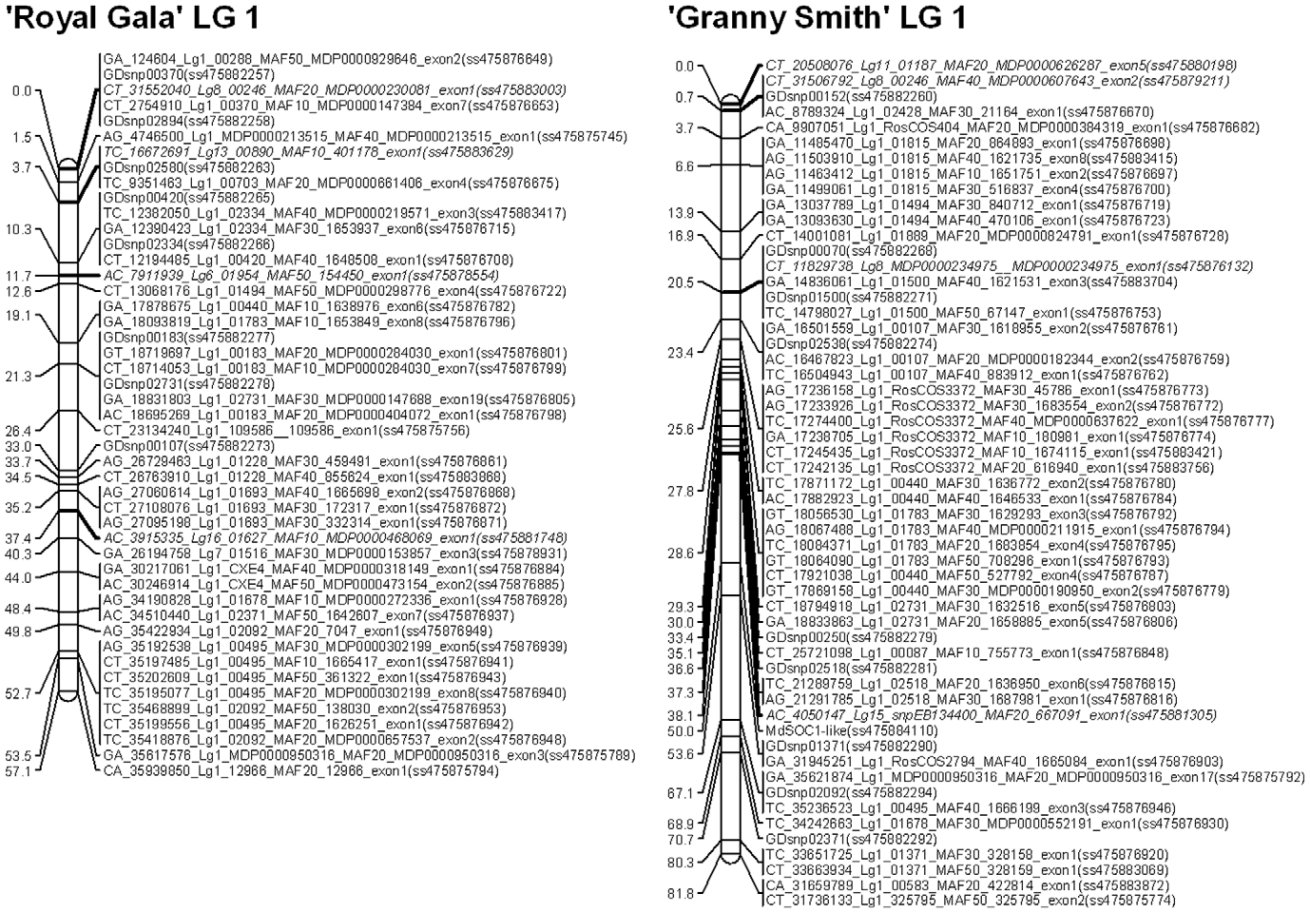
Genetic maps of linkage group (LG) 1 constructed using the ‘Royal Gala’×‘Granny Smith’ segregating population. Marker names indicate the type of SNP, physical location on the ‘Golden Delicious’ genome assembly (in base pair), cluster marker, minor allelic frequency (MAF) in the 27 re-sequenced accessions, gene model, position in the predicted gene model, and NCBI dbSNP accession number (in parentheses).

Evaluation of the IRSC apple Infinium® II 8K array using 1619 individuals, including individual accessions and segregating populations, yielded 7692 successful beadtypes (97.7%) of which 5554 (72.2%) were polymorphic (Table 4). The remaining 2138 SNPs (27.8%) exhibited poor quality genotype clustering or were monomorphic. Numbers of polymorphic SNPs per LG ranged between 519 and 215 on LG2 and LG7, respectively and the number of clusters per LG varied according to LG genetic length and ranged from 62 to 108. Polymorphic SNPs were observed for 1190 (93.7%) of the attempted clusters. The GenomeStudio® *GenTrain* scores for the polymorphic SNPs ranged from 0.35 to 0.92 and the call frequency ranged from 0.73 to 1. In total, 1061 polymorphic SNPs had a very low MAF of between 0 and 0.05, while 3371 polymorphic SNPs had MAF>0.05 with a call frequency >0.95 and reliability score (50% GC) >0.4. Of the previously validated GDsnps and GoldenGate®-validated SNPs, 629 (90.7%) and 55 (74.3%), respectively, were polymorphic. In the ‘Royal Gala’×‘Granny Smith’ segregating population, 2810 SNPs in 994 clusters were polymorphic. Of these, 2223 SNPs (79.2%) were heterozygous for one parent and homozygous for the other (pseudo-testcross configuration) and the remaining 587 (20.8%) were heterozygous for both parents. Polymorphic pseudo-testcross SNPs in this population were successfully used as independent markers for linkage map construction (Figure 4). The average distance between markers was 0.88 cM and 0.91 cM for ‘Royal Gala’ and ‘Granny Smith’, respectively, and the largest gap between informative clusters was 24.3 cM for ‘Royal Gala’ and 25 cM for ‘Granny Smith’. However, the marker density could be increased in these large gaps by adding markers heterozygous for both parents.

**Figure 5:**
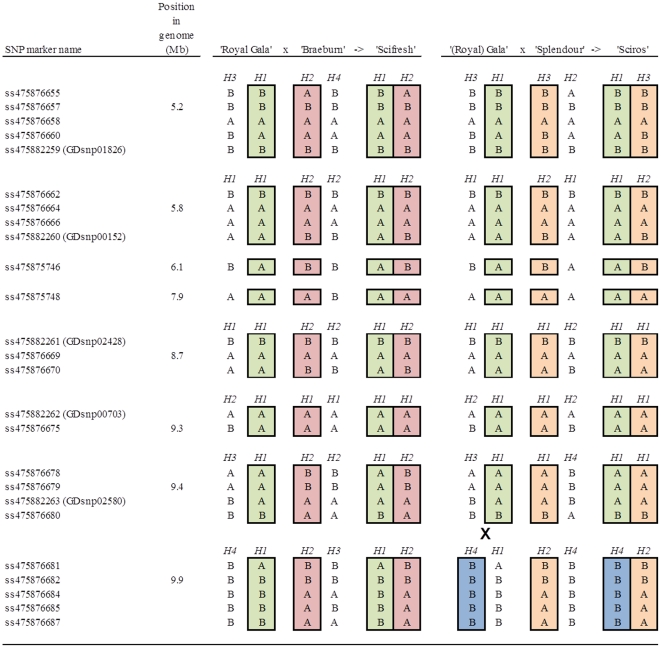
Example of the usefulness of the International RosBREED SNP Consortium (IRSC) apple 8K SNP array v1 for developing haplotypes. SNP markers were the same as represented in the example of Figure 3. Haplotypes inferred using FlexQTL™ for each cluster of SNPs are numbered from *H1* to *H4*). Haplotypes inherited by the progeny from the parents are boxed and colored coded for each parent. A putative recombination is indicated by a cross for the ‘(Royal) Gala’×‘Splendour’−>‘Sciros’ trio.

The small region of LG1 presented in Figure 3 spanned 25 polymorphic markers in two trios with both parents and one progeny (Figure 5): ‘Royal Gala’×‘Braeburn’−>‘Scifresh’ and ‘(Royal) Gala’×‘Splendour’−>‘Sciros’. The haplotypes inherited from each parent could be inferred for the eight SNP clusters, with three of the clusters having four haplotypes. One putative recombination event was detected in the ‘(Royal) Gala’×‘Splendour’−>‘Sciros’ trio between the second last and last two clusters.

## Discussion

### A SNP-based strategy for apple genome scanning

An international community of apple genomics, genetics, and breeding researchers recently united with the goal of developing high-resolution genome scanning capability for germplasm of cultivated apple. The resulting medium to high-throughput multiplexed SNP assay presented and evaluated here is the culmination of a collective technical effort and shared resources that would have been beyond the means of any single entity. This publicly available genomics resource will provide unprecedented resolution for discovery of marker-locus-trait association discovery, for description of the genetic architecture of quantitative traits, for investigation of genetic variation (neutral and functional), and will enable genomic selection in apple. To ensure maximum utility, the apple SNP array involved several key design features.

To ensure that genome scans using the SNP array relate directly to the draft apple genome sequence of [11], priority was given to including on the array previously validated and genetically mapped SNPs from ‘Golden Delicious’ that had been used to anchor the genetic and physical maps of apple. GDsnps also provided internal controls for validation of SNPs with the GoldenGate® assay. The series of previously validated GDsnps [7], [11] as a whole performed better than the new series of SNPs in both the GoldenGate® assay and the apple Infinium® 8K array evaluations (100% and 90.7% success rates, respectively). This result underscores that prior validation is useful when developing a high-throughput SNP array, to minimize the proportion of attempted SNPs that are not informative. Future SNP arrays for apple will be firmly based on the SNPs validated here with the first apple Infinium® array.

Genetic and physical distribution of SNPs across the apple genome was also a strong consideration in array design. The whole genome sequence of apple [11] was a critical resource that greatly increased the likelihood that the final array would cover the majority of the apple genome. Organizing each SNP according to genetic and physical location enabled efficient genome saturation, helped to avoid redundancy and spanned gaps that would have occurred if a collection of random SNPs had been employed. Attention was given to ends of chromosomes; however, not beyond the edges of a genetically defined linkage group. Our genetic positioning of focal points relied on the assembly of the apple genome sequence into linkage groups by [11] that involved anchoring of metacontigs to a genetic map comprising 1643 SNP loci. Errors in this assembly will reduce the effectiveness of the IRSC apple 8K SNP array v1 to span the apple genome genetically. As the number of high quality (MAF>0.05, *p50GC* >0.4, call rate >0.95) polymorphic markers provided by the array developed here is greater than that was used for the genome assembly (3371 vs. 1643), the new set of SNPs from the array will be useful for improvement of the apple genome assembly.

A haplotype-targeting strategy was adopted to maximize information gained using the SNP array and to decrease computation time for mapping software. Clusters of multiple SNPs were spaced at 1 cM intervals (equivalent to an average of 446.2 kb, [11]) rather than evenly spreading independent loci (approximately every 0.17 cM or 75 Kb). As bi-allelic SNPs are often less informative than co-dominant multi-allelic markers such as SSRs, we clustered four to 10 SNP markers within a small, essentially non-recombining, physical distance, i.e., ±50 Kb around focal points. Individual SNPs within clusters were spaced at a minimum of 2 Kb to reduce redundancy of haplotype capture. This strategy will enable combination of the information from individual SNPs into haplotypes that will provide fully informative and easy to handle multi-allelic markers and capture all unique haplotypes within genotyped germplasm. A preliminary analysis using two parent-child trios indicated that our clustering strategy is capable of facilitating the identification of haplotypes within and among SNP clusters. Nevertheless, it was recently observed that the ‘Golden Delicious’ genome assembly contains some erroneous gene position assignments as reported recently for a set of candidate genes for flowering [30] and RosCOS markers [31]. Therefore, it is advisable to perform independent linkage analysis of polymorphic SNPs to ensure that they locate in the expected cluster, to avoid haplotype construction using SNPs that are not truly physically linked. Use of a consensus map of fully informative markers is more powerful for QTL analysis than partially informative markers, as all alleles of each marker locus can be simultaneously evaluated for their phenotypic effects. A highly saturated genome of fully informative markers is also highly amenable to integrated QTL analysis over multiple pedigree-connected families of variable size with the FlexQTL™ software (www.flexqtl.nl), which supports the Pedigree-Based Analysis approach [32]. We chose GDsnps as focal points for clusters because of their established genetic resolution of 1 cM. RosCOS loci were used because their genetic locations were known and they provide an efficient orthologous marker system for the assessment of comparative genome synteny across Rosaceae family genera [24]. Finally, candidate genes were included on the array because of their putative enhanced capacity for association of detected genetic variation with horticultural trait variation.

Our principal source of SNPs was from whole-genome resequencing of apple accessions for which pedigree connections were considered, in order to represent founder contributions to breeding germplasm efficiently and thereby increase the probability of detecting informative haplotypes segregating in breeding programs. Previously available SNP markers for apple were derived from the ‘Golden Delicious’ genome sequence [11] and while ‘Golden Delicious’ is a common founder in pedigrees of cultivated apple, it nevertheless accounts for only a fraction of potentially unique sources of genetic variation among modern apple cultivars [7]. Most accessions in the SNP detection germplasm set were included because they represent the most common founders in the ancestry of cultivated apple. The recent-generation cultivars ‘CrimsonCrisp’ and Co-op 15 have missing links in their ancestry and were included in the sequencing panel to help to capture informative haplotypes in current breeding programs.

To obtain sufficient sequencing depth for each accession, we chose the NGS technique provided by Illumina GA II instrumentation. Because of its high information output and relative affordability, such high-throughput sequencing by synthesis has revolutionized agricultural and horticultural genomics, enabling sequencing of complete genomes of plant species including several crops of the Rosaceae family: apple, peach, and strawberry. Further to these physical mapping successes, sequencing by synthesis has revealed detailed information on sequence polymorphism within species of interest [9], [33], [34], [35], [36].

The sequencing of ‘Cox's Orange Pippin’ at two locations enabled us to compare SNP detection in independent samples. We believe that the high proportion of SNPs with non-matching genotypes between the ‘Cox's Orange Pippin’ samples was due to low sequence coverage providing insufficient reads at a locus to support detection of both alleles at heterozygous SNP loci. Two strategies can be used for SNP detection using NGS. Our strategy was to sequence a wide range of cultivated apple accessions at relatively low coverage to capture as many breeding-relevant SNPs as possible. An alternative approach would have been to re-sequence fewer accessions at higher coverage to enable prediction of SNP genotypes more correctly for each accession. However, this latter strategy would have reduced opportunities to identify rare haplotypes carried by some breeding lineages. The sequences of 27 apple accessions generated in this study are an important resource for apple geneticists, providing a comprehensive inventory of point mutations potentially underlying genetic variation for important horticultural traits beyond their use here in development of the 8K SNP array. The strategy of re-sequencing higher numbers of accessions at low coverage proved successful, as shown by evaluation of both the GoldenGate® and Infinium® arrays. While the *in silico* genotypes of the 27 accessions based on whole-genome sequencing are not always correct, most of the predicted SNPs are real and were converted into SNP markers in the final Infinium® II array, to provide more than 5500 polymorphic markers in a diverse breeding germplasm set.

To enhance the capacity to identify and characterize genetic control regions for horticultural traits – a common intended use of the array – hundreds of candidate genes were built into the array design. Many of these candidate genes have the advantage of being well studied by researchers [25], [37], [38], [39], [40], [41], [42], thus providing loci of known or readily interpretable genetic variation of immediate interest to genome scan users and a relatively fast path to practical application. Similarly, the choice of exonic SNPs for the array biases the genome scan to the preferred target of the gene space of apple genomes. Although the bias toward exonic SNPs was initially only a guide based on performance in the GoldenGate® array, the sheer number of SNPs available from the detection step allowed the final design to feature only those SNPs putatively located in exons. However, it is unlikely that the SNP array will span causative mutations for all characters, as such mutations are often located in non-coding regulatory regions, such as the mutation demonstrated to control red pigmentation of the apple fruit flesh and foliage, which is located in the promoter region of *MYB10* [39].

### SNP filtering and validation

A set of 7867 apple SNPs corresponding to 1355 clusters was employed to construct the IRSC apple 8K SNP array v1. These SNPs were chosen from more than 2 million SNPs detected across the apple genome, utilizing 67 Gb of NGS data from 27 apple accessions. The number of SNPs detected was more than 250 times as many as needed for array construction, allowing very fastidious filtering. Firstly, some SNP types were filtered out because of the choice of the technology for the final array, with C/G and A/T transversions being rejected as they require two Infinium® II probes for detection. Further decisions on the filtering criteria used to develop the final SNP array were made on the basis of empirically determined thresholds obtained from a GoldenGate® validation assay of a representative subset of 144 SNPs, in order to optimize the success rate for screening apple breeding germplasm. The success rate in the validation assay varied according to the type of SNPs and their parameters.

The main reason for choosing exonic SNPs for construction of the Infinium® II array was the observation that randomly distributed SNPs tended to perform more poorly in the GoldenGate® than SNPs located within coding regions. This imbalance in performance might be explained by a lower number of nucleotide polymorphisms in exonic regions, increasing stringency of hybridization between the array features and target genomic DNA. The presence of undetected SNPs in the sequences flanking genes has been observed to reduce both the SNP success rate [43], [44] and the rate of SNP conversion [12]. This finding represents a major concern for those developing high-throughput SNP screening tools in highly heterozygous genomes, such as the forest tree species genomes [12], [43]. Based on recent reports, apple is among the most genetically polymorphic agricultural species analyzed to date. For example, [5] estimated a frequency of 1 SNP per 149 bp based on public apple ESTs, while [7] identified an average of 1 SNP per 455 bp in a single cultivar and 1 SNP per 52 bp across germplasm. This can be compared with figures ranging from 1 SNP every 61 bp in maize [45], 1 SNP/117 bp in grapevine (based on the ‘Pinot noir’ genome sequence; [46]) or 1 SNP/64–104 bp (based on multi-locus cultivar analysis [46], [47], to 1 SNP/5,700 bp in japonica rice cultivars [48]. To address this very specific issue, SNPs were discarded from consideration during the filtering step, whenever another SNP was detected within 50 bases. Nevertheless, because of the low sequencing coverage for each of the 27 accessions, it is probable that many flanking SNPs have not been detected.

MAF influenced the success of SNPs in the GoldenGate® assay, where SNPs with low MAF based on the NGS detection were less likely to be polymorphic than SNPs with higher MAF. Our strategy of choosing SNPs exhibiting a range of MAFs within clusters evenly spaced across the apple genome will help to capture available haplotypes with high-MAF SNPs (i.e., MAF>0.4), improving detection of heterozygosity for any assayed germplasm individual, while lower-MAF SNPs (MAF <0.2), tend to come from specific founders and improve identification of rare haplotypes.

### IRSC apple 8K SNP array v1 evaluation

Based on information derived from a consensus apple genetic map of 1343 cM (M. Troggio, unpublished), the IRSC apple 8K SNP array v1 corresponds to an average density of one focal point every 1 cM. Overall, the IRSC apple 8K SNP array v1 was successful, as 5554 markers were polymorphic in the examined germplasm set, with 4368 SNPs having a MAF>0.05, and 3371 having a high call rate, reliability score, and MAF. While this result indicates that at least 3371 markers are likely to provide a genotype when the array is used for screening any germplasm set, any given genetic mapping experiment can expect to obtain ∼4000 polymorphic bi-allelic markers or ∼1000 fully informative haplotypes. The number of SNPs in the array that can successfully provide a genotype might be improved by enhancing DNA quality of the template used for the experiment. In our array evaluation, the set of accessions tested was from a breeding program where the number of individuals was maximized at the expense of the DNA quality.

We have demonstrated that the array is effective for use in genetic mapping, providing 994 focal points in a ‘Royal Gala’×‘Granny Smith’ segregating population, a sufficient number for the construction of a highly saturated genetic map. This resolution will be also sufficient to implement genomic selection in apple [29]. However, the resolution will not be high enough for association mapping studies using unrelated germplasm, because a recent study indicates that linkage disequilibrium among apple cultivars decays faster than this, based on calculations in a apple germplasm collection [7].

### Conclusion

The International RosBREED SNP Consortium apple 8K SNP array v1 has been developed for public use by apple geneticists worldwide. The design and evaluation of the array has indicated that it will be effective for a wide range of germplasm and applications such as high-resolution genetic mapping, QTL detection and characterization, marker-assisted introgression, and genomic selection.

### Genomic resources

All SNPs detected, the SNPs chosen for the IRSC apple Infinium® II 8K array, and the GenomeStudio cluster file developed are deposited in the Genome Database for Rosaceae (www.rosaceae.org). SNPs are available in dbSNP (http://www.ncbi.nlm.nih.gov/projects/SNP/) under accessions ss475875741 to ss475892397.

## Supporting Information

Table S1 The 160 apple accessions used for the GoldenGate® SNP validation assay.
(DOCX)

Table S2 List of 7867 apple SNPs on the Apple Infinium® II array v1. The NCBI dbSNP accession, location on the ‘Golden Delicious’ genome assembly [11], and source of SNP are indicated.
(XLS)

## References

[1] Peace CP, Norelli JL, Folta K, Gardiner S (2009). Genomics approaches to crop improvement in Rosaceae.. Genetics and Genomics of Rosaceae.

[2] Liebhard R, Gianfranceschi L, Koller B, Ryder CD, Tarchini R (2002). Development and characterisation of 140 new microsatellites in apple (*Malus*×*domestica* Borkh.).. Molecular Breeding.

[3] Silfverberg-Dilworth E, Matasci CL, Van de Weg WE, Van Kaauwen MPW, Walser M (2006). Microsatellite markers spanning the apple (*Malus*×*domestica* Borkh.) genome.. Tree Genetics & Genomes.

[4] Wang A, Aldwinckle H, Forsline P, Main D, Fazio G (2011). EST contig-based SSR linkage maps for *Malus*×*domestica* cv Royal Gala and an apple scab resistant accession of *M. sieversii*, the progenitor species of domestic apple.. Molecular Breeding.

[5] Chagné D, Gasic K, Crowhurst RN, Han Y, Bassett HC (2008). Development of a set of SNP markers present in expressed genes of the apple.. Genomics.

[6] Han Y, Chagné D, Gasic K, Rikkerink EHA, Beever JE (2009). BAC-end sequence-based SNPs and Bin mapping for rapid integration of physical and genetic maps in apple.. Genomics.

[7] Micheletti D, Troggio M, Zharkikh A, Costa F, Malnoy M (2011). Genetic diversity of the genus *Malus* and implications for linkage mapping with SNPs.. Tree Genetics & Genomes.

[8] Wittwer CT, Reed GH, Gundry CN, Vandersteen JG, Pryor RJ (2003). High-resolution genotyping by amplicon melting analysis using LCGreen.. Clinical Chemistry.

[9] Huang X, Wei X, Sang T, Zhao Q, Feng Q (2010). Genome-wide association studies of 14 agronomic traits in rice landraces.. Nature Genetics.

[10] Myles S, Chia J-M, Hurwitz B, Simon C, Zhong GY (2010). Rapid genomic characterization of the genus *Vitis*.. PloS One.

[11] Velasco R, Zharkikh A, Affourtit J, Dhingra A, Cestaro A (2010). The genome of the domesticated apple (*Malus*×*domestica* Borkh.).. Nature Genetics.

[12] Grattapaglia D, Silva OB, Kirst M, de Lima BM, Faria DA (2011). High-throughput SNP genotyping in the highly heterozygous genome of *Eucalyptus*: assay success, polymorphism and transferability across species.. BMC Plant Biology.

[13] Shen G-Q, Abdullah KG, Wang QK (2009). The TaqMan method for SNP genotyping.. Methods Mol Biol,.

[14] Iezzoni A, Weebadde C, Luby J, Yue CY, Weg Evd (2010). RosBREED: Enabling marker-assisted breeding in Rosaceae.. Acta Horticulturae.

[15] Li RQ, Yu C, Li YR, Lam TW, Yiu SM (2009). SOAP2: an improved ultrafast tool for short read alignment.. Bioinformatics.

[16] Wang J, Li R, Li Y, Fang X, Feng B (2008). Genome resequencing and identification of variations by Illumina Genome Analyzer Reads.. Protocol Exchange.

[17] Newcomb RD, Crowhurst RN, Gleave AP, Rikkerink EHA, Allan AC (2006). Analyses of expressed sequence tags from apple.. Plant Physiology.

[18] Wu TD, Watanabe CK (2005). GMAP: a genomic mapping and alignment program for mRNA and EST sequences.. Bioinformatics.

[19] Maliepaard C, Alston FH, van Arkel G, Brown LM, Chevreau E (1998). Aligning male and female linkage maps of apple (*Malus pumila* Mill.) using multi-allelic markers.. Theoretical and Applied Genetics.

[20] Dyk MMv, Rees DJG, van Dyk MM (2009). Bin mapping of EST-SSRs in apple (*Malus*×*domestica* Borkh.).. Acta Horticulturae.

[21] Celton JM, Tustin DS, Chagne D, Gardiner SE (2009). Construction of a dense genetic linkage map for apple rootstocks using SSRs developed from *Malus* ESTs and *Pyrus* genomic sequences.. Tree Genetics & Genomes.

[22] Kenis K, Keulemans J, Davey MW (2008). Identification and stability of QTLs for fruit quality traits in apple.. Tree Genetics & Genomes.

[23] Cabrera A, Kozik A, Howad W, Arus P, Iezzoni AF (2009). Development and bin mapping of a Rosaceae Conserved Ortholog Set (COS) of markers.. BMC Genomics.

[24] Illa E, Sargent DJ, Girona EL, Bushakra J, Cestaro A (2011). Comparative analysis of rosaceous genomes and the reconstruction of a putative ancestral genome for the family.. BMC Evolutionary Biology.

[25] Giovannoni J (2001). Molecular biology of fruit maturation and ripening.. Annual Review of Plant Physiology and Plant Molecular Biology.

[26] Mouradov A, Cremer F, Coupland G (2002). Control of flowering time: Interacting pathways as a basis for diversity.. Plant Cell.

[27] Wang Y, Li J (2008). Molecular basis of plant architecture.. Annual Review of Plant Biology.

[28] Rowan DD, Hunt MB, Dimouro A, Alspach PA, Weskett R (2009). Profiling fruit volatiles in the progeny of a ‘Royal Gala’×‘Granny Smith’ apple (*Malus*×*domestica*) cross.. Journal of Agricultural and Food Chemistry.

[29] Kumar S, Bink M, Volz R, Bus V, Chagné D (2011). Towards genomic selection in apple (*Malus*×*domestica* Borkh.) breeding programmes: Prospects, challenges and strategies.. Tree Genetics & Genomes.

[30] Guitton B, Kelner J, Velasco R, Gardiner S, Chagné D (2011). Genetic control of biennial bearing in apple.. Journal of Experimental Botany.

[31] Bushakra J, Sargent D, Cabrera A, Crowhurst R, Lopez Girona E (2011). Assessing genome synteny between *Malus* and *Fragaria* using Rosaceae conserved orthologous set (RosCOS) markers.. Tree Genetics & Genomes.

[32] Van de Weg WE, Voorrips RE, Finkers R, Kodde LP, Jansen J (2004). Pedigree genotyping: a new pedigree-based approach of QTL identification and allele mining.. Acta Horticulturae.

[33] Lam HM, Xu X, Liu X, Chen WB, Yang GH (2010). Resequencing of 31 wild and cultivated soybean genomes identifies patterns of genetic diversity and selection.. Nature Genetics.

[34] Myles S, Boyko AR, Owens CL, Brown PJ, Grassi F (2011). Genetic structure and domestication history of the grape.. Proceedings of the National Academy of Sciences of the United States of America.

[35] Nelson JC, Wang S, Wu Y, Li X, Antony G (2011). Single-nucleotide polymorphism discovery by high-throughput sequencing in sorghum.. BMC Genomics.

[36] Nordborg M, Weigel D (2008). Next-generation genetics in plants.. Nature.

[37] Costa F, Stella S, Van de Weg WE, Guerra W, Cecchinel M (2005). Role of the genes *Md-ACO1* and *Md-ACS1* in ethylene production and shelf life of apple (*Malus domestica* Borkh).. Euphytica.

[38] Costa F, Van de Weg WE, Stella S, Dondini L, Pratesi D (2008). Map position and functional allelic diversity of *Md-Exp7*, a new putative expansin gene associated with fruit softening in apple (*Malus*×*domestica* Borkh.) and pear (*Pyrus communis*).. Tree Genetics & Genomes.

[39] Espley RV, Brendolise C, Chagne D, Kutty-Amma S, Green S (2009). Multiple repeats of a promoter segment causes transcription factor autoregulation in red apples.. Plant Cell.

[40] Espley RV, Hellens RP, Putterill J, Stevenson DE, Kutty-Amma S (2007). Red colouration in apple fruit is due to the activity of the MYB transcription factor, *MdMYB10*.. Plant Journal.

[41] Gao ZS, van de Weg WE, Schaart JG, Schouten HJ, Tran DH (2005). Genomic cloning and linkage mapping of the *Mal d 1* (PR-10) gene family in apple (*Malus domestica*).. Theoretical and Applied Genetics.

[42] Wang A, Yamakake J, Kudo H, Wakasa Y, Hatsuyama Y (2009). Null mutation of the *MdACS3* gene, coding for a ripening-specific 1-Aminocyclopropane-1-Carboxylate Synthase, leads to long shelf life in apple fruit.. Plant Physiology.

[43] Eckert AJ, Pande B, Ersoz ES, Wright MH, Rashbrook VK (2009). High-throughput genotyping and mapping of single nucleotide polymorphisms in loblolly pine (*Pinus taeda* L.).. Tree Genetics & Genomes.

[44] Pindo M, Vezzulli S, Coppola G, Cartwright DA, Zharkikh A (2008). SNP high-throughput screening in grapevine using the SNPlexTM genotyping system.. BMC Plant Biology.

[45] Krzywinski M, Schein J, Birol I, Connors J, Gascoyne R (2009). Circos: An information aesthetic for comparative genomics.. Genome Research.

[46] Lijavetzky D, Antonio Cabezas J, Ibanez A, Rodriguez V, Martinez-Zapater JM (2007). High throughput SNP discovery and genotyping in grapevine (*Vitis vinifera* L.) by combining a re-sequencing approach and SNPlex technology.. BMC Genomics.

[47] Vezzulli S, Micheletti D, Riaz S, Pindo M, Viola R (2008). A SNP transferability survey within the genus *Vitis*.. BMC Plant Biology.

[48] Yamamoto T, Nagasaki H, Yonemaru J-i, Ebana K, Nakajima M (2010). Fine definition of the pedigree haplotypes of closely related rice cultivars by means of genome-wide discovery of single-nucleotide polymorphisms.. BMC Genomics.

