# Supplementary material for: Genome-Wide SNP Detection, Validation, and Development of an 8K SNP Array for Apple
Source: PLoS One. 2012 Feb 21;7(2):e31745. doi: 10.1371/journal.pone.0031745 (PMC3283661; doi:10.1371/journal.pone.0031745)
Supplement: Table S1 — The 160 apple accessions used for the GoldenGate® SNP validation assay. (DOCX) [file pone.0031745.s001.docx]

**Table S1**

| Designation | Parent 1 | Parent 2 |
| --- | --- | --- |
| ‘Akane’ | ‘Jonathan’ | ‘Worcester Pearmain’ |
| ‘Ambrosia’ | ‘Golden Delicious’ | ‘Delicious’ |
| ‘Anna’ | - | ‘Golden Delicious’ |
| ‘Arlet’ | ‘Golden Delicious’ | ‘Idared’ |
| ‘Aurora Golden Gala’ | ‘Gala’ | ‘Splendour’ |
| ‘Autumn Crisp’ | ‘Golden Delicious’ | ‘Monroe’ |
| ‘Beacon’ | ‘Malinda’ | - |
| ‘Beauty of Bath’ | - | - |
| ‘Ben Davis’ | - | - |
| ‘Blushing Golden’ | - | - |
| ‘Braeburn’ | - | - |
| ‘Cameo’ | ‘Golden Delicious’ | ‘Delicious’ |
| ‘Co-op 15’ | NJ27 | PRI 612-1 |
| ‘Co-op 17’ | Ill#2 | PRI 668-100 |
| ‘Cox’s Orange Pippin’ | Ribston Pippin | Blenheim Orange |
| ‘CrimsonCrisp’ | PCFW2-134 | PRI 669-205 |
| ‘Cripp’s Red’ | ‘Golden Delicious’ | ‘Lady Williams’ |
| ‘Cripp’s Pink’ | ‘Golden Delicious’ | ‘Lady Williams’ |
| ‘Dayton’ | NJ123249 | PRI 1235-100 |
| ‘Delblush’ | ‘Golden Delicious’ | ‘Blushing Golden’ |
| ‘Delicious’ | - | - |
| ‘Delorgue’ | ‘Delcorf’ | - |
| ‘Dolgo’ | - | - |
| ‘Duchess of Oldenburg’ | - | - |
| ‘Elstar’ | ‘Golden Delicious’ | ‘Ingrid Marie’ |
| ‘Empire’ | ‘McIntosh’ | ‘Delicious’ |
| ‘Enterprise’ | PRI 1661-2 | PRI 1661-1 |
| ‘Esopus Spitzenburg’ | - | - |
| F_2_-26829-2-2 | - | - |
| ‘Fiesta’ | ‘Cox’s Orange Pippin’ | ‘Idared’ |
| ‘Frostbite’ | - | - |
| ‘Fuji’ | ‘Ralls Janet’ | ‘Red Delicious’ |
| ‘Gala’ | ‘Kidd’s Orange Red’ | ‘Golden Delicious’ |
| ‘Geneva’ | - | - |
| ‘Ginger Gold’ | ‘Golden Delicious’ | - |
| GMAL 4327 | *M. sieversii* | - |
| GMAL 4328 | *M. sieversii* | - |
| GMAL 4329 | *M. sieversii* | - |
| GMAL 4332 | *M. sieversii* | - |
| ‘Golden Delicious’ | - | - |
| ‘Goldrush’ | ‘Golden Delicious’ | ‘Co-op 17’ |
| ‘Goodland’ | ‘Patten Greening’ | - |
| ‘Granny Smith’ | - | - |
| ‘Haralson’ | ‘Malinda’ | ‘Wealthy’ |
| ‘Hatsuaki’ | ‘Jonathan’ | ‘Golden Delicious’ |
| ‘Honeycrisp’ | ‘Keepsake’ | - |
| ‘Hudson’ | - | - |
| ‘Idared’ | ‘Jonathan’ | ‘Wagener’ |
| ‘Ingrid Marie’ | ‘Cox’s Orange Pippin’ | - |
| ‘James Grieve’ | ‘Cox’s Orange Pippin’ | - |
| ‘Jonafree’ | PRI 855-102 | NJ31 |
| ‘Jonathan’ | ‘Esopus Spitzenburg’ | - |
| ‘Keepsake’ | ‘Frostbite’ | ‘Northern Spy’ |
| ‘Kerr’ | ‘Dolgo’ | ‘Haralson’ |
| ‘Kidd’s Orange Red’ | ‘Delicious’ | ‘Cox’s Orange Pippin’ |
| ‘Lady Williams’ | - | - |
| ‘Linda’ | ‘Langford Beauty’ | - |
| *M. floribunda* clone 821 | - | - |
| ‘Macoun’ | ‘McIntosh’ | ‘Jersey Black’ |
| ‘Malinda’ | - | - |
| ‘Mantet’ | ‘Tetofsky’ | - |
| ‘McIntosh’ | - | - |
| MN 1691 | ‘Fireside’ | ‘Goodland’ |
| MN 1702 | - | - |
| MN 1706 | - | - |
| MN 1760 | 74-6-77 | - |
| MN 1764 | 74-6-77 | - |
| MN 1802 | - | - |
| MN 1837 | MN1627 | MN1691 |
| MN 1839 | MN1627 | ‘Prima’ |
| MN 1888 | MN1627 | MN1691 |
| MN 1915 | ‘Sweet 16’ | - |
| ‘Monark’ | - | - |
| ‘Montgomery’ | - | - |
| ‘Nicola’ | ‘Splendour’ | ‘Gala’ |
| NJ 90 | NJ136055 | ‘Spartan’ |
| ‘Northern Spy’ | - | - |
| NY 03 | ‘Autumn Crisp’ | ‘Fuji’ |
| NY 241 | ‘Honeycrisp’ | NY 752 |
| NY 543 | - | - |
| NY 632 | - | ‘Splendour’ |
| NY 752 | ‘Golden Delicious’ | NY88 |
| NY 913 | - | ‘Ginger Gold’ |
| NY 92607-16 | - | - |
| ‘Pinova’ | ‘Clivia’ | ‘Golden Delicious’ |
| ‘Pitmaston Pineapple’ | - | - |
| ‘Pixie Crunch’ | PCFW2-134 | PRI669-205 |
| ‘Prima’ | PRI14-510 | NJ123249 |
| ‘Ralls Janet’ | - | - |
| ‘Red Dougherty’ | - | - |
| ‘Regent’ | - | - |
| ‘Rome Beauty’ | - | - |
| ‘Russian Seedling’ | - | - |
| ‘Sawa’ | ‘Fantazja’ | ‘Primula’ |
| ‘Scifresh’ | ‘Royal Gala’ | ‘Braeburn’ |
| ‘Sciglo’ | ‘Gala’ | ‘Splendour’ |
| ‘Sciros’ | ‘Gala’ | ‘Splendour’ |
| ‘Silken’ | ‘Honeygold’ | ‘Sunrise’ |
| ‘SnowSweet’ | ‘Sharon’ | ‘Fireside’ |
| ‘Sonya’ | ‘Gala’ | ‘Delicious’ |
| ‘Spartan’ | ‘McIntosh’ | - |
| ‘Splendour’ | - | - |
| ‘Sundance’ | ‘Golden Delicious’ | PRI 1050-201 |
| ‘Sunrise’ | - | PCF 3-120 |
| ‘Sweet 16’ | ‘Frostbite’ | ‘Northern Spy’ |
| ‘Telamon’ | ‘McIntosh’ | ‘Golden Delicious’ |
| ‘Tsugaru’ | ‘Golden Delicious’ | ‘Jonathan’ |
| ‘Wagener’ | - | - |
| ‘Wealthy’ | - | - |
| ‘Williams’ | ‘Jolana’ | - |
| ‘Winesap’ | - | - |
| ‘Winter Banana’ | - | - |
| ‘Worcester Pearmain’ | - | - |
| WSU 2 | ‘Splendour’ | ‘Gala’ |
| WSU 5 | ‘Splendour’ | ‘Co-op 15’ |
| WSU 7 | NJ 90 | ‘Goldrush’ |
| ‘Yellow Newtown Pippin’ | - | - |
| ‘Yellow Transparent’ | - | - |
| ‘Zestar’ | ‘State Fair’ | MN 1691 |
| AJ79 | ‘Malling 9’ | ‘Robusta 5’ |
| AJ140 | ‘Malling 9’ | ‘Robusta 5’ |
| AJ146 | ‘Malling 9’ | ‘Robusta 5’ |
| AJ154 | ‘Malling 9’ | ‘Robusta 5’ |
| AJ155 | ‘Malling 9’ | ‘Robusta 5’ |
| AJ162 | ‘Malling 9’ | ‘Robusta 5’ |
| AJ170 | ‘Malling 9’ | ‘Robusta 5’ |
| AJ172 | ‘Malling 9’ | ‘Robusta 5’ |
| EB244 | ‘Gala’ | ‘Braeburn’ |
| EB308 | ‘Gala’ | ‘Braeburn’ |
| EB417 | ‘Gala’ | ‘Braeburn’ |
| EB464 | ‘Gala’ | ‘Braeburn’ |
| EB481 | ‘Gala’ | ‘Braeburn’ |
| EB518 | ‘Gala’ | ‘Braeburn’ |
| EB651 | ‘Gala’ | ‘Braeburn’ |
| EB831 | ‘Gala’ | ‘Braeburn’ |
| GDxA_11 | ‘Golden Delicious’ | ‘Anna’ |
| GDxA_12 | ‘Golden Delicious’ | ‘Anna’ |
| GDxA_19 | ‘Golden Delicious’ | ‘Anna’ |
| GDxA_51 | ‘Golden Delicious’ | ‘Anna’ |
| GDxA_52 | ‘Golden Delicious’ | ‘Anna’ |
| GDxA_276 | ‘Golden Delicious’ | ‘Anna’ |
| GDxA_335 | ‘Golden Delicious’ | ‘Anna’ |
| GDxA_353 | ‘Golden Delicious’ | ‘Anna’ |
| PxF_9 | ‘Prima’ | ‘Fiesta’ |
| PxF_19 | ‘Prima’ | ‘Fiesta’ |
| PxF_22 | ‘Prima’ | ‘Fiesta’ |
| PxF_31 | ‘Prima’ | ‘Fiesta’ |
| PxF_34 | ‘Prima’ | ‘Fiesta’ |
| PxF_35 | ‘Prima’ | ‘Fiesta’ |
| PxF_39 | ‘Prima’ | ‘Fiesta’ |
| PxF_40 | ‘Prima’ | ‘Fiesta’ |
| PxF_42 | ‘Prima’ | ‘Fiesta’ |
| TelxBB_19 | ‘Telamon’ | ‘Braeburn’ |
| TelxBB_20 | ‘Telamon’ | ‘Braeburn’ |
| TelxBB_21 | ‘Telamon’ | ‘Braeburn’ |
| TelxBB_22 | ‘Telamon’ | ‘Braeburn’ |
| TelxBB_23 | ‘Telamon’ | ‘Braeburn’ |
| TelxBB_24 | ‘Telamon’ | ‘Braeburn’ |
| TelxBB_25 | ‘Telamon’ | ‘Braeburn’ |
| TelxBB_26 | ‘Telamon’ | ‘Braeburn’ |
